# Supplementary material for: Enhanced spin Seebeck effect via oxygen manipulation
Source: Nat Commun. 2023 Jun 8;14:3365. doi: 10.1038/s41467-023-39116-x (PMC10250387; doi:10.1038/s41467-023-39116-x)
Supplement: Supplementary file 1 — Supplementary Information [file 41467_2023_39116_MOESM1_ESM.pdf]

Supplementary Information

Enhanced spin Seebeck effect via oxygen manipulation

Jeong-Mok Kim, Seok-Jong Kim, Min-Gu Kang, Jong-Guk Choi, Soogil Lee, Jaehyeon Park, Cao Van Phuoc, Kyoung-Whan Kim, Kab-Jin Kim, Jong-Ryul Jeong, Kyung-Jin Lee and Byong-Guk Park

– Contents –

Note 1. Theoretical models for SSE

Note 2. Numerical calculation of SSE

Note 3. Magnetic moment changes upon heat treatment

Note 4. Gate voltage control of thermoelectric voltage with Pt, Ta electrodes.

Note 5. Thermoelectric voltage (V_{TE}) with thicker CoFeB.

Note 6. Estimation of shunting effect

Note 7. Effect of oxidized FM lattices with larger thermal gradient

Supplementary Note 1. Theoretical models for SSE

1-a. Magnon temperature model

We describe the magnon temperature model[S1-S4] for an insulator/FM1/FM2/NM structure where the exchange interaction J_{ex} differs between FM1 and FM2 (**Fig. S1a**, the same as Fig. 1c of the main text). Following Ref. [S4], the equivalent thermal circuit is shown in **Fig. S1b**. Here Q_1^{FM} (Q_2^{FM}) is the magnon heat current of FM1 (FM2), R_1^{FM} (R_2^{FM}) is the magnon heat resistance of FM1 (FM2), and $R_{\mathrm{F2|F1}}^{\mathrm{int}}$ ($R_{\mathrm{F1|N}}^{\mathrm{int}}$) is the interface magnon heat resistance at the FM2/FM1 (FM1/NM) interface. Note that the temperature difference between magnons and electrons at the thermal grounds is zero[S4].

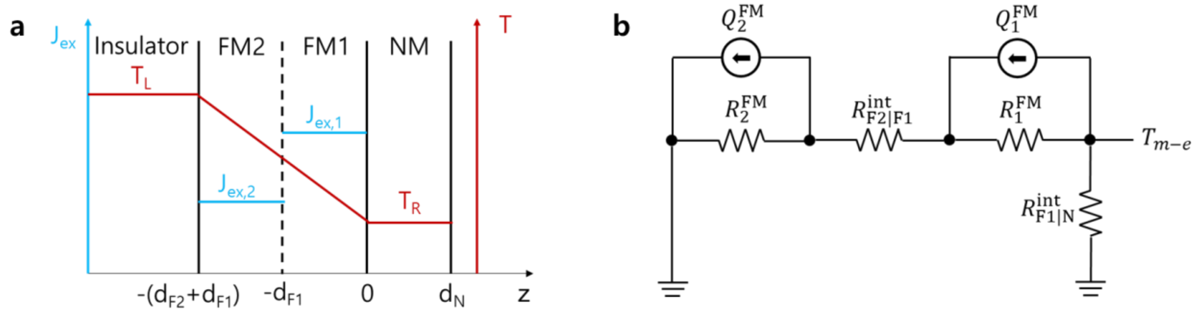

Figure S1| Magnon temperature model. a, Model structure where FM consists of two ferromagnetic layers with different exchange interaction J_{ex}. **b**, The thermal circuit equivalent to the layer structure of **a**.

From the thermal circuit model, we obtain

$$T_{m-e} = \frac{Q_1^{\mathrm{FM}} R_1^{\mathrm{FM}} + Q_2^{\mathrm{FM}} R_2^{\mathrm{FM}}}{R_1^{\mathrm{FM}} + R_2^{\mathrm{FM}} + R_{\mathrm{F2|F1}}^{\mathrm{int}} + R_{\mathrm{F1|N}}^{\mathrm{int}}} R_{\mathrm{F1|N}}^{\mathrm{int}}, \quad \text{(S1)}$$

which creates a spin accumulation in NM and thus V_{SSE} through ISHE. To obtain the ratio r_{SSE1} of V_{SSE} with $J_{ex,1} \neq J_{ex,2}$ to V_{SSE} with $J_{ex,1} = J_{ex,2}$, we use the following assumptions. We first assume no loss of magnon heat current at the FM2|FM1 interface (i.e.,

$R^{\rm int}_{\rm F2/F1} \to \infty$), leading to

$$T_{m-e} = \frac{Q_1^{\rm FM} R_1^{\rm FM} + Q_2^{\rm FM} R_2^{\rm FM}}{R_1^{\rm FM} + R_2^{\rm FM} + R^{\rm int}_{\rm F1|N}} R^{\rm int}_{\rm F1|N}, \tag{S2}$$

We further simplify Eq. (S2) by taking the limit of $R_1^{\rm FM} + R_2^{\rm FM} \gg R^{\rm int}_{\rm F1|N}$, yielding

$$T_{m-e} = \frac{Q_1^{\rm FM} R_1^{\rm FM} + Q_2^{\rm FM} R_2^{\rm FM}}{R_1^{\rm FM} + R_2^{\rm FM}} R^{\rm int}_{\rm F1|N}. \tag{S3}$$

Note that the other limit (i.e., $R_1^{\rm FM} + R_2^{\rm FM} \ll R^{\rm int}_{\rm F1|N}$) gives $T_{m-e} = Q_1^{\rm FM} R_1^{\rm FM} + Q_2^{\rm FM} R_2^{\rm FM} = d_{F1} + d_{F2}$ [because $Q_i^{\rm FM} = \kappa_i \nabla T$ and $R_i^{\rm FM} = d_{Fi}/(\kappa_i A)$], which is independent of any magnetic properties and thus irrelevant to our work. Using $\kappa_i \propto (J_{ex,i})^{-1/2}$ [S4] (Supplementary Table I), Eq. (S3) gives the ratio $r_{\rm SSE1}$ as

$$r_{\rm SSE1} = \frac{d_{F1} + d_{F2}}{d_{F1} + d_{F2}\sqrt{\frac{J_{ex,1}}{J_{ex,2}}}}, \tag{S4}$$

which is Eq. (3) in the main text.

1-b. Magnon drift-diffusion model

We describe the magnon drift-diffusion model[S4,S5] for the same structure of **Fig. S1**. In FMi (i = 1, 2), a set of drift-diffusion equation for the magnon chemical potential μ_i^m and the magnon spin current j_i^m is given by[S4]

$$\frac{\partial \mu_i^m}{\partial z^2} = \frac{\mu_i^m}{l_{Fi}^2}, \tag{S5}$$

$$j_i^m = -\frac{\sigma_{Fi}}{\hbar}\frac{\partial \mu_i^m}{\partial z} - L_i \nabla T, \tag{S6}$$

where l_{Fi}, σ_{Fi}, and L_i are the magnon diffusion length, the magnon spin conductivity, the spin Seebeck coefficient of FMi, respectively. In NM, a set of drift-diffusion equation for the spin chemical potential μ^s and the spin current j^s is given by[S4]

$$\frac{\partial\mu^s}{\partial z^2} = \frac{\mu^s}{l_N^2}, \quad \text{(S7)}$$

$$j^s = -\frac{\hbar}{2e}\left(\frac{\sigma}{2e}\frac{\partial\mu^s}{\partial z} + \sigma_{SH}E\right), \quad \text{(S8)}$$

where l_N, σ, σ_{SH}, and E are the spin diffusion length, the charge conductivity, the spin Hall conductivity of NM and the electric field, respectively. The boundary conditions at outer boundaries are: $j_2^m(z = -d_{F2} - d_{F1}) = j^s(z = d_N) = 0$. The boundary conditions at the FM2/FM1 interface ($z = -d_{F1}$) are: $j_2^m = j_1^m$ and $j_2^m = G_{\mathrm{F2|F1}}(\mu_2^m - \mu_1^m)$, where $G_{\mathrm{F2|F1}}$ is the interfacial magnon conductance at this interface. The boundary conditions at the FM1/NM interface ($z = 0$) are: $j_1^m = j^s$ and $j_1^m = G_{\mathrm{F1|N}}(\mu_1^m - \mu^s)$, where $G_{\mathrm{F1|N}}$ is the interfacial spin conductance at this interface.

Solving the drift-diffusion equations [Eqs. (S5)-(S8)] with the boundary conditions gives V_{SSE} as

$$V_{\mathrm{SSE}} = \theta_{SH}\nabla T\,\frac{2el_N d[(d_{F1}^2 l_{F2}^2 L_1 + d_{F2}^2 l_{F1}^2 L_2)\sigma_{F1} + 2d_{F1}d_{F2}l_{F1}^2 L_1\sigma_{F2}]}{\hbar d_N l_{F1}^2 l_{F2}^2 \sigma\sigma_{F1}}\operatorname{csch}\frac{d_N}{l_N}\sinh^2\frac{d_N}{2l_N}, \quad \text{(S9)}$$

where θ_{SH} $(= \sigma_{SH}/\sigma)$ is the spin Hall angle of NM and d is the electrode distance to measure V_{SSE}. We obtain Eq. (4) with assumptions of continuous magnon chemical potential at the FM2/FM1 interface (i.e., $G_{\mathrm{F2|F1}} \to \infty$) and $d_{F1(2)} \ll l_{F1(2)}$ to simplify V_{SSE}. Given $\sigma_F \propto L \propto J_{ex}^{-1/2}$ and $l_F \propto J_{ex}^{1/2}$ [S4] (Supplementary Table I), the ratio r_{SSE2} of V_{SSE} with $J_{ex,1} \neq J_{ex,2}$ to V_{SSE} with $J_{ex,1} = J_{ex,2}$ for the magnon drift-diffusion model is given as

$$r_{\mathrm{SSE2}} = \left[1 + \left(\left(\frac{d_{F2}}{d_{F1}}\right)^2 + 2\frac{d_{F2}}{d_{F1}}\right)\left(\frac{J_{ex,1}}{J_{ex,2}}\right)^{\frac{3}{2}}\right]\left(1 + \frac{d_{F2}}{d_{F1}}\right)^{-2}, \quad \text{(S10)}$$

which is Eq. (5) in the main text.

Supplementary Table 1. Dependence of magnon transport coefficients on exchange interaction J_{ex} [S4]

	Symbol	Expression	Dependence on J_{ex}
Magnon thermal de Broglie length	Λ	$\sqrt{4\pi J_{ex}/(k_B T)}$	$J_{ex}^{1/2}$
Magnon heat conductivity	κ	$\frac{35\zeta(7/2) J_{ex} k_B^2 T\tau}{2\hbar^2\Lambda^3}$	$J_{ex}^{-1/2}$
Magnon spin conductivity	σ_F	$\frac{4\zeta(3/2)^2 J_{ex}\tau}{\hbar^2\Lambda^3}$	$J_{ex}^{-1/2}$
Spin Seebeck coefficient	L	$\frac{10\zeta(5/2) e J_{ex} k_B T\tau}{\hbar^2\Lambda^3}$	$J_{ex}^{-1/2}$
Magnon spin diffusion length	l_F	$\frac{\sqrt{8 J_{ex} k_B T\tau\tau_{mr}/3}}{\hbar}$	$J_{ex}^{1/2}$

Here, τ_{mr} is the magnon relaxation time related to the Gilbert damping and τ is the total relaxation time including all scattering processes.

Supplementary Note 2. Numerical calculation of SSE

We use Landau-Lifshitz-Gilbert (LLG) equation for i-th magnetic moment $\hat{\mathbf{m}}_i$ in 1-D chain along the *z* axis,

$$\frac{d\hat{\mathbf{m}}_i}{dt} = -\gamma\hat{\mathbf{m}}_i \times \left(\mathbf{H}_{\mathrm{eff},i} + \mathbf{H}_{\mathrm{th},i}\right) + \alpha_i\hat{\mathbf{m}}_i \times \frac{d\hat{\mathbf{m}}_i}{dt}, \quad \text{(S11)}$$

where γ is the gyromagnetic ratio and α_i is the damping parameter of the i-th lattice. The effective field of the i-th lattice is $\mathbf{H}_{\mathrm{eff},i} = \frac{2A_{ex,i}}{M_s}(\hat{\mathbf{m}}_{i-1} + \hat{\mathbf{m}}_{i+1}) - \frac{2K_h}{M_s}m_z\hat{\mathbf{z}} + H_{ext}\hat{\mathbf{x}}$, which consists of the exchange field, easy-plane(xy plane) anisotropy field, and external magnetic field along the +*x* axis. $A_{ex,i}$ is the exchange stiffness of the i-th lattice, M_s is the saturation magnetization, K_h is the easy-plane anisotropy energy. The thermal fluctuation field $\mathbf{H}_{\mathrm{th},i}$ obeys the Gaussian ensemble[S6],

$$\langle \mathbf{H}_{\mathrm{th},i}(t) \rangle = 0, \quad \text{(S12)}$$

$$\langle \mathbf{H}_{\mathrm{th},i}(t)\mathbf{H}_{\mathrm{th},j}(t') \rangle = \frac{2k_B T_i \alpha_i}{\gamma V M_s}\delta_{ij}\delta(t - t'), \quad \text{(S13)}$$

where k_B is the Boltzmann constant, T_i is the temperature of the i-th lattice and V is the volume of the ferromagnet unit cell.

To calculate the SSE, we apply a numerical procedure of Ref. [S7]. We calculate the time-average of spin pumping current of the i-th lattice, proportional to $\langle [\hat{\mathbf{m}}_i \times d\hat{\mathbf{m}}_i/dt]_x \rangle$. The system consists of 10 ferromagnetic lattices. $\langle [\hat{\mathbf{m}}_i \times d\hat{\mathbf{m}}_i/dt]_x \rangle$ is calculated for 11 different temperature distributions: One is for the linear temperature gradient from 1 K (1st lattice) to 10 K (10th lattice), for which the $\langle [\hat{\mathbf{m}}_i \times d\hat{\mathbf{m}}_i/dt]_x \rangle$ of i-th lattice is defined as $j_{Tgrad}(i)$. The other 10 cases are with a constant temperature from 1 K to 10 K, for which the $\langle [\hat{\mathbf{m}}_i \times d\hat{\mathbf{m}}_i/dt]_x \rangle$ of i-th lattice at a constant i K is defined as $j_{Tconst}(i)$. Then the thermal

spin pumping current $j_{s,1}$ at the i-th lattice is defined as $j_{s,i} = j_{Tgrad}(i) - j_{Tconst}(i)$. As shown in Fig. 2a of the main text, the normal metal layer is in contact to an atomic ferromagnetic layer with the lowest temperature. Therefore, $j_{s,1}$ is the thermal spin pumping current injected to the normal metal, which we focus below.

Oxidation of magnetic materials induces a reduction of Curie temperature and an increase of damping[S8]. Reduction of the Curie temperature can be considered as reduction of the exchange stiffness constant. In simulations, therefore, we consider inhomogeneous exchange stiffness or damping within the 1-D atomic chain consisting of 10 lattices. We assume 3 lattices at the hotter region are oxidized and consider the exchange stiffness ($A_{ex,ox}$) or damping parameters (α_{ox}) different from the other 7 lattices (i.e., non-oxidized lattices). Non-oxidized lattices have fixed exchange stiffness and damping parameter as $A_{ex,0} = 2 \times 10^{-7}$ erg cm^{-1} and $\alpha_0 = 0.01$. We vary these parameters at oxidized lattices as $0.05A_{ex,0} \leq A_{ex,ox} \leq 2A_{ex,0}$ and $0.05\alpha_0 \leq \alpha_{ox} \leq 6\alpha_0$.

Fig. S2a shows the result with varying damping parameter in oxidized lattices. $J_{s,homo}$ is $j_{s,1}$ for the homogeneous case (i.e., $\alpha_{ox} = \alpha_0$) whereas $J_{s,inhomo}$ is $j_{s,1}$ for the inhomogeneous case (i.e., $\alpha_{ox} \neq \alpha_0$). The ratio $J_{s,inhomo}/J_{s,homo}$ as a function of α_{ox}/α_0 is plotted in the figure. We find that the spin pumping current from the SSE is enhanced (reduced) when the oxidized lattices have a larger (smaller) damping parameter than non-oxidized lattices. This tendency can be understood by the fluctuation-dissipation theorem stating that the amount of fluctuations must be balanced by the amount of dissipation. In our case, this theorem gives the proportionality of the thermal fluctuation field H_{th} to $\sqrt{T_i\alpha_i}$. Therefore, an enhanced damping acts like an enhanced temperature for the thermal fluctuations of magnetization. As a result, the enhanced damping in the oxidized region (i.e., the hotter region) increases an

effective temperature gradient, which in turn increases the thermal spin pumping current.

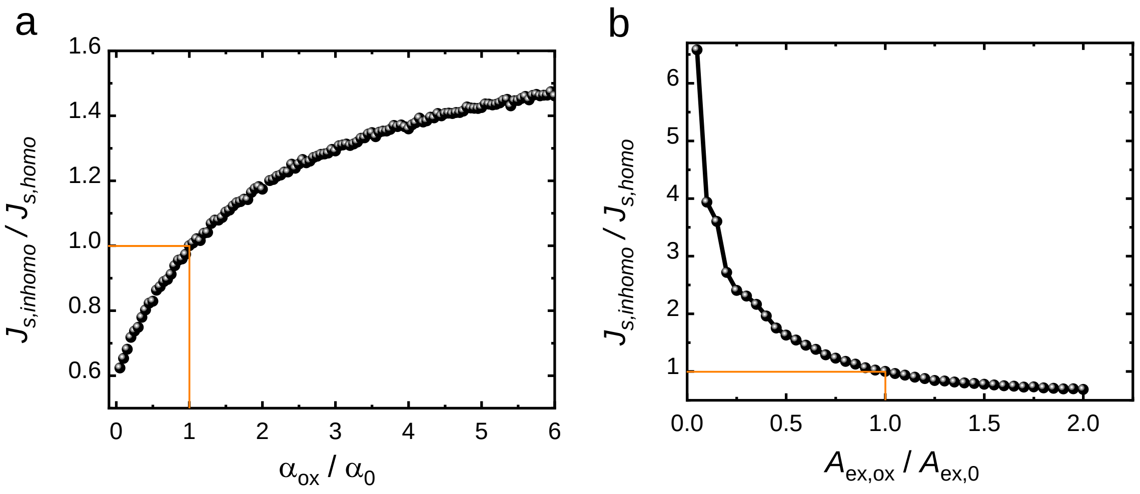

Figure S2| The ratio $J_{s,inhomo}/J_{s,homo}$ with varying **a,** the damping parameter and **b,** the exchange stiffness constant in oxidized lattices. Parameters for simulations are: $M_s =$ 1000 emu cm^{-3}, $\gamma = 1.76 \times 10^7$ Oe^{-1}s^{-1}, $K_h = 4 \times 10^6$ erg cm^{-3}, and $H_{ext} = 100$ Oe.

Fig. S2b shows $J_{s,inhomo}/J_{s,homo}$ with varying the exchange stiffness constant in oxidized lattices. The spin current from the SSE is enhanced (reduced) when the oxidized lattices have a smaller (larger) exchange stiffness than non-oxidized lattices. Instead of the true thermal gradient, the gradient of the exchange stiffness constant can induce an effective thermal gradient. The number of thermal magnons follows the Bloch $T^{3/2}$ law, where the fractional change of magnetization is proportional to $(k_B T/A_{ex})^{3/2}$. When $A_{ex,ox} < A_{ex,0}$, additional thermal magnons induced by the exchange gradient flow toward non-oxidized lattices from the hotter region to the colder region, resulting in an enhanced spin pumping current.

The results of **Fig. S2** show that both a larger damping parameter and a smaller exchange stiffness in the oxidized lattices enhance the spin pumping current from SSE. However, the

damping modulation enhances the spin pumping current only by ~50% even for $\alpha_{ox} = 6\alpha_0$ (**Fig. S2a**), which is insufficient to explain the experimental observation. On the other hand, the exchange modulation shows a much larger enhancement of the spin pumping current than the damping modulation (**Fig. S2b**). Therefore, we attribute the large change in SSE of the experiment to the exchange modulation by oxidation.

The oxidation may also result in changes of the anisotropy or the saturation magnetization. To check whether these changes can explain our experimental observation, we perform LLG simulation with varying one of these two magnetic properties while fixing other properties (**Fig. S3**). In this simulation, non-oxidized FM lattices have fixed easy-plane anisotropy energy ($K_{h,0} = 4 \times 10^6$ erg cm^{-3}) and saturation magnetization ($M_{s,0} = 1000$ emu cm^{-3}), while we vary these parameters in oxidized FM lattices as $-2\,K_{h,0} \leq K_{h,ox} \leq 2K_{h,0}$ for Fig. S3a and $0.05M_{s,0} \leq M_{s,ox} \leq M_{s,0}$ for Fig. S3b. In **Fig. S3a**, a negative $K_{h,ox}$ means a perpendicular anisotropy of the oxidized FM lattices, assuming interfacial perpendicular anisotropy induced by an adjacent oxide layer. In **Fig. S3b**, consider the cases with $M_{s,ox}$ smaller than $M_{s,0}$ since the oxidation of magnetic materials induces a reduction of the Curie temperature, resulting in a reduction of the saturation magnetization.

Figure S3a shows the result with various $K_{h,ox}$ in oxidized FM lattices. The spin pumping current is found to change within 10 %, compared to that of the homogeneous anisotropy ($K_{h,ox} = K_{h,0}$) case. Therefore, the anisotropy change due to oxidation has no noticeable effect on the SSE. This tendency can be understood by the dominance of exchange interaction over inhomogeneous anisotropy energy in thermal spin pumping. **Figure S3b** shows the result with various $M_{s,ox}$ in oxidized FM lattices. The simulation shows that the spin pumping current is reduced when the oxidized FM lattices have a smaller saturation magnetization than non-

oxidized FM lattices. It is a contrary tendency from the experiment. We understand this tendency as a consequence of an enhanced effective exchange field ($H_{ex} = 2A_{ex}/M_s$) in the oxidized FM lattices. Therefore, varying the anisotropy energy or the saturation magnetization of oxidized FM lattices is unable to describe a largely enhanced SSE due to the oxidation.

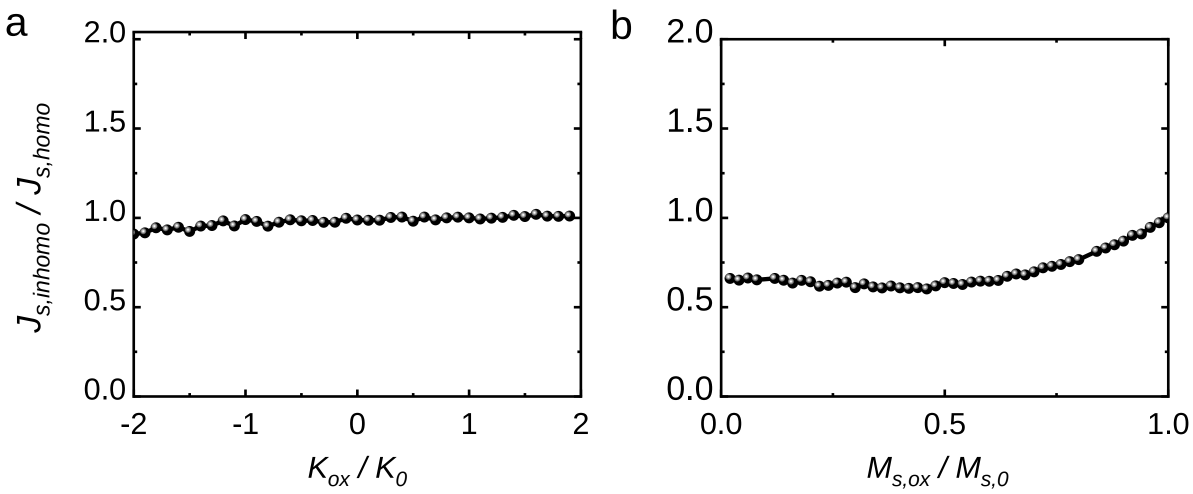

Figure S3| The ratio $J_{s,inhomo}/J_{s,homo}$ with varying **a,** anisotropy energy and **b,** the saturation magnetization in oxidized FM lattices. Parameters: $M_{s,0} = 1000$ emu cm^{-3}, $K_{h,0} = 4 \times 10^6$ erg cm^{-3}, $\alpha_i = 0.01$, $A_{ex,i} = 2 \times 10^{-7}$ erg cm^{-1}, $H_{ext} = 100$ Oe, and the unit cell volume = $1000 \times 1000 \times 0.4$ nm^3.

The oxidation of magnetic materials could also result in a formation of an antiferromagnetic (AFM) phase such as CoO and FeO_x at the interface[S8]. Therefore, we calculate the spin pumping current for the case that the oxidized lattices have an AFM exchange interaction ($A_{ex,0} = -2 \times 10^{-7}$ erg cm^{-1}). **Fig. S4a** shows the schematic of SSE calculation with AFM exchange interaction. For 10-lattice system, we vary the number of lattices having the AFM exchange. Each calculation is normalized by $J_{s,homo}$ with no AFM interaction ($= J_{s,FM}$). **Fig.**

S4b shows that the formation of an AFM phase at the hotter region does not enhance the spin pumping current. When the system is fully an AFM phase (the number of AFM couplings = 9), the spin pumping current is found to significantly decrease. This decrease is not meaningful because it originates from the spin-flop transition in the calculation. In summary, a possible formation of AFM phase due to the oxidation cannot explain the experimental observation.

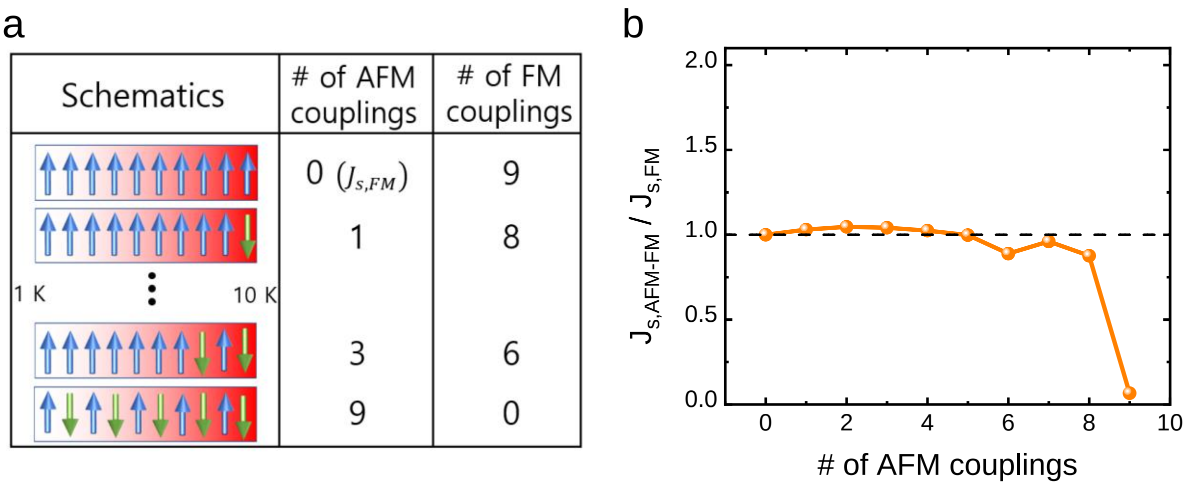

Figure S4| a, The schematic of SSE calculation with antiferromagnetic interactions. The sum of antiferromagnetic (AFM) and ferromagnetic (FM) couplings is always 9, and we calculate SSE with increasing the number of AFM couplings of the system starting from the lattice with 10 K (the hotter region). All the SSE results are normalized with $J_{s,FM}$, SSE result with no antiferromagnetic coupling. **b,** Normalized SSE result with varying antiferromagnetic interactions.

Supplementary Note 3. Magnetic moment changes upon heat treatment

To check that the oxygen migration occurs during the measurement of the temperature-dependent R_H, we measured the magnetic moment of the sample before and after heat treatment at 380 K. We used two samples of a W(4 nm)/CoFeB(2 nm)/AlO_x(1.5 nm) structure with plasma oxidation times of 0 s and 150 s. **Figures S5 a,b** show the results that the magnetic moments of the samples do not change upon the heat treatment regardless of the plasma oxidation time. This result allows us to rule out the effect of oxygen migration on the temperature dependence of R_H.

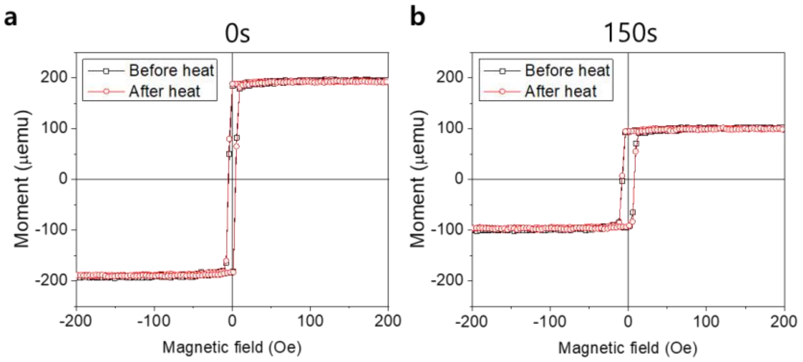

Figure S5| a, b Magnetic moment as a function of magnetic field in a W (4 nm)/CoFeB (2 nm)/AlO_x (2 nm) structure with different plasma oxidation time; **a**. 0 s and **b**.150 s, where the samples are heated for 5 minutes at 380 K.

Supplementary Note 4. Gate voltage control of thermoelectric voltage with Pt, Ta electrodes.

We fabricated a Pt, Ta (3 nm)/CoFeB (2 nm)/AlO_x (2 nm) structure and examined the V_G-induced ΔV_{TE}. Because of the positive spin Hall angle of Pt, the SSE voltage of this sample is expected to be opposite to that of the sample with W (**Figure S6a**). V_{TE} was measured while rotating a magnetic field of 100 mT in the *x*-*y* plane (azimuthal angle φ_B). **Figure S6b** shows that the magnitude of ΔV_{TE} of the Pt/CoFeB sample increases when V_G = – 13 V and decreases when V_G = + 13 V. Since the SSE has the same sign as the anomalous Nernst effect (ANE) in the Pt/CoFeB sample with a positive spin Hall angle, the increase in ΔV_{TE} indicates that the magnitude of the SSE in the Pt/CoFeB sample increases at the negative V_G. This is consistent with the results of the W/CoFeB sample. We also checked a Ta/CoFeB sample with a negative spin Hall angle of Ta. **Figure S6c** shows the angle-dependent V_{TE} of the Ta/CoFeB sample with V_G = ± 13 V; ΔV_{TE} decreases when a V_G of −13V is applied, the same as for the W/CoFeB sample. Finally, we compare the V_G-induced ΔV_{TE} between the samples using the V_G modulation efficiency $\xi = [\Delta V_{TE}(V_G = +13V) - \Delta V_{TE}(V_G = -13V)]/R$, where R is the sample resistance. **Figure S6d** plots the ξ value for each sample along with the spin Hall angle of the non-magnetic electrodes[S9,S10]. This demonstrates that the ξ is closely related to the magnitude and sign of the spin Hall angle. These results corroborate that the V_G modulates the SSE in the NM/CoFeB structures.

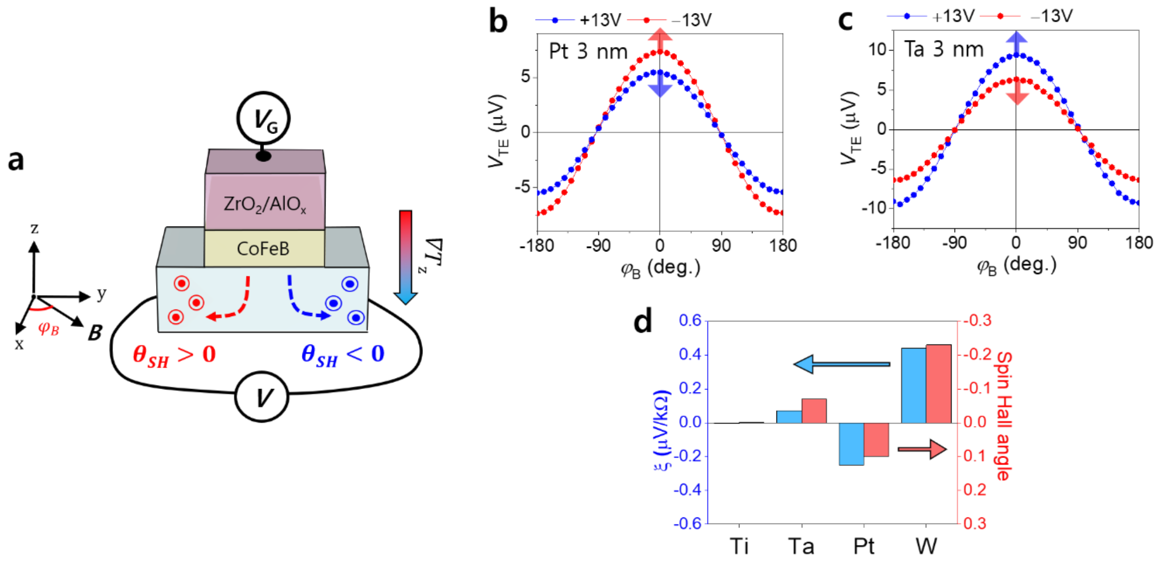

Figure S6| a Schematic of a V_G modulation of thermoelectric voltage (V_{TE}) depending on the NM materials with different spin Hall angle (θ_{SH}) in a NM (3 nm)/CoFeB (2 nm)/AlO_x (2 nm) structure. **b-c**, V_{TE} as a function of magnetic field angle (φ_B) at $V_G = +13$ V (blue), −13 V (red) for a Pt/CoFeB/AlO_x (b) and Ta/CoFeB/AlO_x (c) structure. **d,** The V_G modulation efficiency (ξ) of NM (3 nm)/CoFeB (2 nm)/AlO_x (2 nm) samples along with spin Hall angle of NM. Here, NM is Ti, Ta, Pt, and W.

Supplementary Note 5. Thermoelectric voltage (V_{TE}) with thicker CoFeB.

To check if the SSE voltage increases with CoFeB thickness, we fabricated a sample of a Pt (3 nm)/CoFeB (6 nm)/AlO_x (1.5 nm) structure and measured thermoelectric voltage (V_{TE}). Note that we use a Pt electrode with a positive spin Hall angle that makes the ANE and SSE have the same sign in the Pt/CoFeB structure, allowing us to directly compare the magnitude of V_{TE} (ΔV_{TE}) between the samples with different CoFeB thicknesses. **Figure S7a** shows V_{TE} of the Pt (3 nm)/CoFeB (6 nm)/AlO_x (1.5 nm) structure while sweeping an in-plane magnetic field (B_x). The ΔV_{TE} value of the sample is ~ 8.7 μV, which is much larger than that of the Pt (3 nm)/CoFeB (2 nm)/AlO_x (1.5 nm) structure (~ 5.4 μV). In addition, we found that the ΔV_{TE} magnitude is further enhanced by increasing the plasma oxidation time from 100 s to 400 s (**Figure S7b**), which is consistent with the results in Figure 3 of the main text. We confirm that the main result of our study, the enhancement of TE signal by interfacial oxidation, also works for samples with thicker ferromagnetic materials.

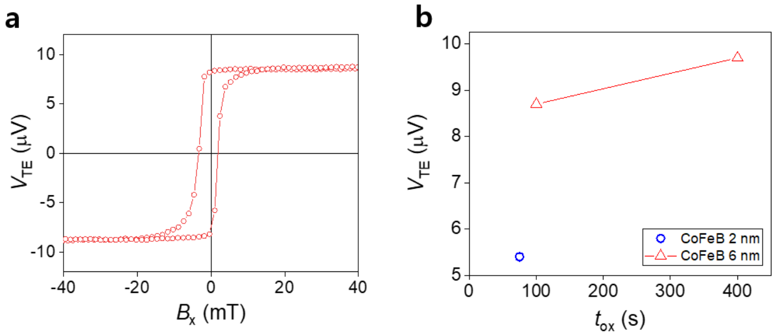

Figure S7| a, V_{TE} a function of magnetic field (B_x) in a Pt (3 nm)/CoFeB (6 nm)/AlO_x (1.5 nm) structure with a plasma oxidation of 100 s. **b,** V_{TE} as a function of the plasma oxidation time t_{ox} in Pt (3 nm)/CoFeB (6 nm)/AlO_x (1.5 nm) structures. The blue circle represents a sample with t_{CoFeB} = 2 nm.

Supplementary Note 6. Estimation of shunting effect

To examine the shunting effect, we first measured the resistivities of the W and CoFeB layers. **Figure S8a** shows the reciprocal of resistance (R) normalized by the length (L) and width (w) of the sample as a function of the CoFeB thickness (t_{CoFeB}) in the W (4 nm)/CoFeB (t_{CoFeB})/AlO_x (2 nm) structures. From the y-intercept and slope of the linear fit of the graph, the resistivities of W and CoFeB were extracted as 216 μΩ·cm and 160 μΩ·cm, respectively. We next measured the temperature dependence of R in the W (4 nm)/CoFeB (1 nm)/AlO_x (2 nm) structure with different V_G of ± 13 V. **Figure S8b** shows that the sample with V_G = −13 V has a larger R than that with +13 V, which holds over the entire measurement temperature range from 20 K to 380 K. This indicates that more CoFeB is oxidized when negative V_G is applied. Note that the weak temperature dependence of R is attributed to the amorphous characteristic of β tungsten[S11]. Assuming that the voltage-induced change in R occurs only in the CoFeB layer, we calculated the shunting (S_W) through the W layer of the W(4 nm)/CoFeB (2nm) sample using a parallel circuit model, $S_W = \frac{R_W{}^{-1}}{R_W{}^{-1}+R_{CoFeB}{}^{-1}}$. Here, $R_{W(CoFeB)}$ is the resistance of the W (CoFeB) layer. The S_W value is ~0.62 at V_G = +13 V and increases to ~0.64 at V_G = -13 V. The increasing S_W can increase the SSE (decrease the ANE) in the W/CoFeB sample which corresponds to the 3.2% change of V_{TE} by shunting effect.

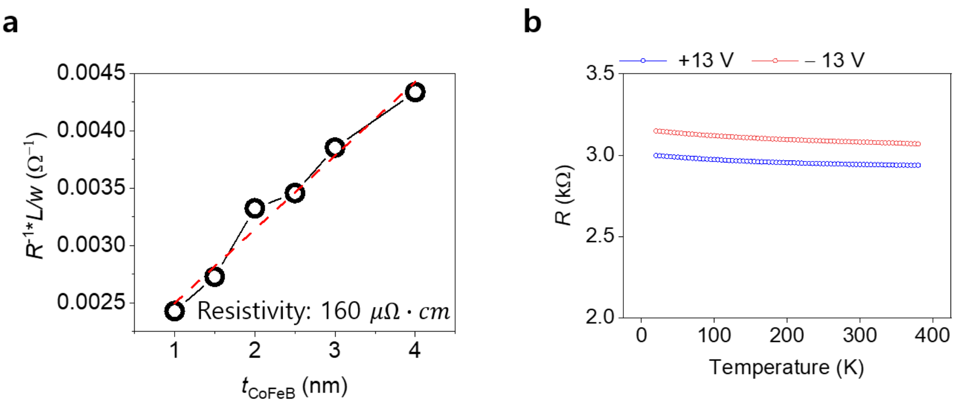

Figure S8| a, $R^{-1}L/w$ as a function of the t_{CoFeB} **b,** Temperature dependence of resistance (R) in W (4 nm)/CoFeB (1 nm)/AlO_x (2 nm) structure with V_G of ± 13 V.

Supplementary Note 7. Effect of oxidized FM lattices with larger thermal gradient

The contribution of heat transfer from electrons and phonons to SSE cannot be ignored, particularly given that insulators typically have lower thermal conductivity compared to metals. This results in a larger thermal gradient for oxidized FM lattices compared to non-oxidized ones. Consequently, the FM oxidation leads to an increase in total SSE not only due to the exchange modulation but also due to the enhanced thermal gradient.

In order to find out which one between the exchange modulation and the enhanced thermal gradient is dominant for the total SSE signal of our experiment, we carry out the following analysis. First, we estimate the SSE enhancement by oxidation from experimental results. Both ANE voltage V_{ANE} and SSE voltage V_{SSE} contribute to net thermoelectric voltage V_{TE}. They are related by a parallel circuit model[S12];

$$\frac{V_{\mathrm{TE}}}{R} = \frac{V_{\mathrm{ANE}}}{R_{\mathrm{F}}} + \frac{V_{\mathrm{SSE}}}{R_{\mathrm{N}}}, \qquad \text{(S14)}$$

where R [$= 1/(R_{\mathrm{F}}^{-1} + R_{\mathrm{N}}^{-1})$] is the total resistance of FM/normal metal (NM) bilayer and R_{F} (R_{N}) is the resistance of FM (NM).

We estimate V_{ANE} from V_{TE} data of Ti (3 nm)/CoFeB (2 nm)/$\mathrm{AlO_x}$ sample assuming no SSE contribution to V_{TE}. It is because this sample has a negligible V_{SSE} [i.e., a small spin Hall angle of Ti and almost no change in V_{TE} at the gate voltage of ±13 V (see Fig. 2f of main text)]. The resistivity of CoFeB is measured to be 160 $\mu\Omega\cdot\mathrm{cm}$ (**Fig. S9a**), which gives R_{CoFeB} = 53.3 kΩ (device width w = 15 μm and device length L = 1,000 μm). From the measured total resistance ($R_{\mathrm{Ti/CoFeB}}$ = 38.0 kΩ), we obtain R_{Ti} = 132.2 kΩ. Using Eq. (S14) and the measured V_{TE} (= 13.7 μV; Fig. 2f of main text), we then obtain V_{ANE} of 19.2 μV.

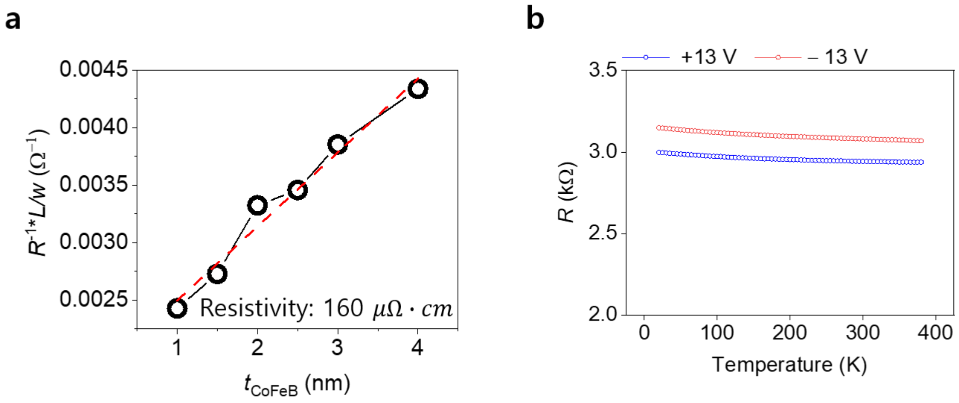

Figure S9 | a, $R^{-1}L/w$ as a function of t_{CoFeB} (device width w = 15 μm, device length L = 1,000 μm). **b,** Temperature dependence of resistance (R) in W (4 nm)/CoFeB (1 nm)/AlO_x (2 nm) structure with V_G of ± 13 V (device width w = 5 μm, device length L = 35 μm).

The gate voltage (V_G) induced SSE enhancement of W (4 nm)/CoFeB (2 nm)/AlO_x sample is estimated as follows. R_W is calculated to be 32.0 kΩ using the measured total resistance ($R_{W/CoFeB}$ = 20.0 kΩ) and R_{CoFeB} = 53.3 kΩ. Using Eq. (S14) and V_{TE} of W/CoFeB/AlOx sample with $V_G = +13$ V (Fig. 2c of main text), we obtain V_{SSE} at $V_G = +13$ V to be -2.7 μV, which corresponds to the SSE without oxidation. On the other hand, using the same procedure, we obtain V_{SSE} at $V_G = -13$ V to be -18.4 μV, which is enhanced by oxidation. Then, the SSE enhancement ratio by oxidation is about 5.8 [= (18.4-2.7)/2.7]. It is noted that we do not consider the oxidation induced reduction in the CoFeB thickness for this estimation. When we consider this reduction (not shown), the SSE enhancement ratio becomes 5.3. Therefore, the oxidation of CoFeB results in the SSE enhancement by about five times.

Next, we estimate the SSE enhancement by an increased thermal gradient of oxidized FM lattices. By comparing the resistances of W-based sample at $V_G = \pm 13$ V (Fig. S9b; device width w = 5 μm, device length L = 35 μm), we estimate the thickness of oxidized CoFeB to be 0.17 nm. We use the heat transfer module of the COMSOL software to calculate the

temperature profile of Ru(20)/ZrO_2(40)/AlO_x(2)/CoFeB(2)/W(4)/SiO_2(100)/SiO in **Fig. S10a** and Ru(20)/ZrO_2(40)/AlO_x(2)/CoFeB-oxidized(0.17)/CoFeB(1.83)/W(4)/SiO_2(100)/SiO in **Fig. 10Sb**, where the numbers in parentheses are the thicknesses in the unit of nm. We use parameters of CoO_x for the CoFeB-oxidized layer because we can find all parameters necessary for COMSOL simulation for CoO_x. A monochromatic 5-μm continuous laser beam of 30 mW is applied on the surface of Ru as in our experiment.

From the COMSOL simulation, the thermal gradient of non-oxidized FM (CoFeB) layer is 1.91 K/μm (**Fig. 10Sa**), while the thermal gradient of oxidized FM (CoO_x) layer is 8.31 K/μm (**Fig. 10Sb**). Then the weighted average of thermal gradient for the CoO_x (0.17 nm)/CoFeB (1.83 nm) bilayer is 2.45 K/μm [= (8.31×0.17+1.91×1.83)/2], which gives an increase of thermal gradient by 28 % [= (2.45/1.91-1) ×100], as compared to un-oxidized CoFeB. This value (= 28 %) is far smaller than the net SSE enhancement (> 500 %) estimated above.

We also check a possibility of the formation of oxides (Fe_2O_3, FeO, Fe_3O_4) other than CoO_x. For these Fe-based oxides, we cannot find all parameters necessary for COMSOL simulations so that we estimate the thermal gradient assuming the inverse proportionality of the thermal gradient to the thermal conductivity as this assumption is consistent with our COMSOL simulation [thermal conductivity of CoFeB (κ_{CoFeB})[S13] = 87 W/(m K)$^{-1}$, thermal conductivity of CoO_x ($\kappa_{\mathrm{CoO_x}}$)[S14]= 20 W/(m K)$^{-1}$, calculated thermal gradient of CoFeB (dT_{CoFeB}) = 1.91 K/μm, and calculated thermal gradient of CoO_x ($dT_{\mathrm{CoO_x}}$) = 8.31 K/μm; $\frac{\kappa_{\mathrm{CoFeB}}}{\kappa_{\mathrm{CoO_x}}} \approx \frac{dT_{\mathrm{CoO_x}}}{dT_{\mathrm{CoFeB}}}$]. From literature[S15], we find $\kappa_{\mathrm{Fe_2O_3}} \approx$ 15 W/(m K)$^{-1}$, $\kappa_{\mathrm{FeO}} \approx$ 10 W/(m K)$^{-1}$, and $\kappa_{\mathrm{Fe_3O_4}} \approx$ 6 W/(m K)$^{-1}$. Using the inverse proportionality, these values gives $dT_{\mathrm{Fe_2O_3}} \approx$ 11.1 K/μm, $dT_{\mathrm{FeO}} \approx$ 16.6 K/μm, and $dT_{\mathrm{Fe_3O_4}} \approx$ 27.7 K/μm, respectively. Then the values of weighted

average of thermal gradient are increased by 41 % for Fe_2O_3, 65 % for FeO, and 115 % for Fe_3O_4, respectively. Overall, therefore, the enhancement of SSE due to the increased thermal gradient of oxides is insufficient to describe the net SSE enhancement (> 500 %) estimated from our measurement. This analysis suggests that the exchange modulation is more dominant for the observed SSE enhancement by oxidation than the increased thermal conductivity.

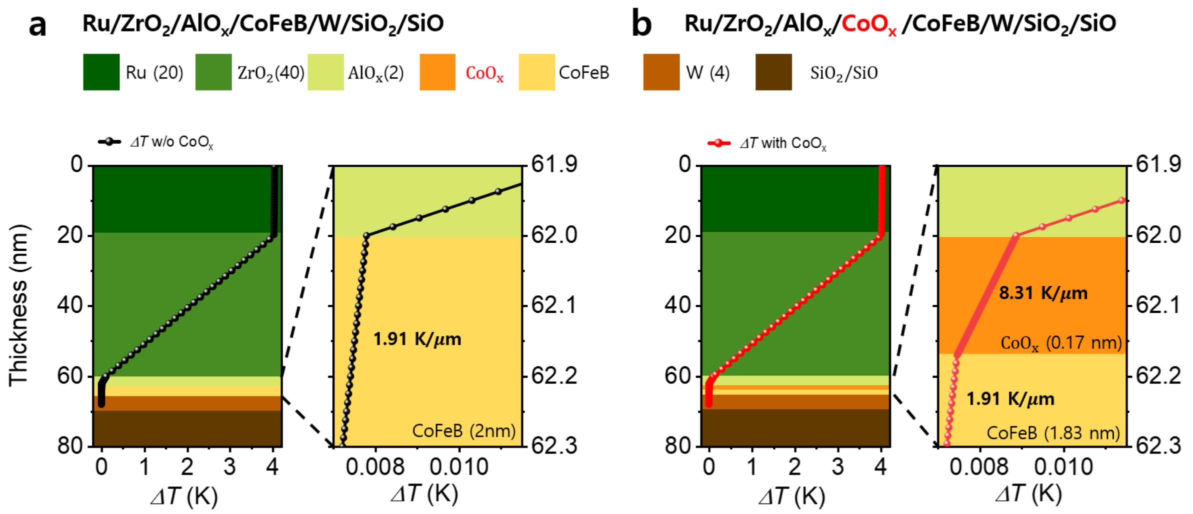

Figure S10| Vertical temperature profiles calculated from COMSOL simulation for **a,** Ru(20 nm)/ZrO_2(40 nm)/AlO_x(2 nm)/CoFeB(2 nm)/W(4 nm)/SiO_2(100 nm)/SiO and **b,** Ru(20 nm)/ZrO_2(40 nm)/AlO_x(2 nm)/CoO_x(0.17 nm)/CoFeB(1.83 nm)/W(4 nm)/SiO_2(100 nm)/SiO samples. Parameters for COMSOL simulation are as follows: Length of sample: 1 mm, width of sample: 15 μm, thickness of substrate= 649.9 μm, laser spot radius = 5 μm, laser power = 30 mW, and total reflectance of sample = 0.77. Thermal conductivities of CoO_x, CoFeB, and AlO_x are 20, 87 and 3.3 W/(m K)$^{-1}$, respectively.

References

[S1] Xiao, J., Bauer, G. E. W., Uchida, K.-i., Saitoh, E. & Maekawa, S. Theory of magnon-driven spin Seebeck effect. *Phys. Rev. B* **81**, 214418 (2010).

[S2] Adachi, H., Ohe, J.-i., Takahashi, S. & Maekawa, S. Linear-response theory of spin Seebeck effect in ferromagnetic insulators. *Phys. Rev. B* **83**, 094410 (2011).

[S3] Schreier, M. *et al*. Magnon, phonon, and electron temperature profiles and the spin Seebeck effect in magnetic insulator/normal metal hybrid structures. *Phys. Rev. B* **88**, 094410 (2013).

[S4] Cornelissen, L. J., Peters, K. J. H., Bauer, G. E. W., Duine, R. A. & van Wees, B. J. Magnon spin transport driven by the magnon chemical potential in a magnetic insulator. *Phys. Rev. B* **94**, 014412 (2016).

[S5] Rezende, S. M. *et al*. Magnon spin-current theory for the longitudinal spin-Seebeck effect. *Phys. Rev. B* **89**, 014416 (2014).

[S6] Brown Jr, W. F. Thermal fluctuations of a single-domain particle. *Phys. Rev.* **130**, 1677 (1963).

[S7] Ohe, J.-i., Adachi, H., Takahashi, S. & Maekawa, S. Numerical study on the spin Seebeck effect. *Phys. Rev. B* **83**, 115118 (2011).

[S8] Lee, D. J. *et al*. Effects of interfacial oxidization on magnetic damping and spin–orbit torques. *ACS Appl. Mater. Interfaces* **13**, 19414 (2021).

[S9] H. L. Wang *et al*. Scaling of Spin Hall Angle in 3*d*, 4*d*, and 5*d* Metals from $Y_3Fe_5O_{12}$/Metal Spin Pumping. *Phys. Rev. Lett.* **197201** (2014).

[S10] J. Liu *et al*. Correlation between the spin Hall angle and the structural phases of early 5*d* transition metals. *Appl. Phys. Lett.* **107**, 232408 (2015).

[S11] Q. Hao *et al*. Beta (β) tungsten thin films: Structure, electron transport, and giant spin Hall effect. *Appl. Phys. Lett.* **106**, 182403 (2015).

[S12] Kim, D.-J. *et al*. Utilization of the antiferromagnetic IrMn electrode in spin thermoelectric devices and their beneficial hybrid for thermopiles. *Adv. Funct. Mater.* **26**, 5507-

5514 (2016).

[S13] Walter, M., *et al*. Seebeck effect in magnetic tunnel junctions. *Nat. mater.* **10**, 742-746 (2011)

[S14] Lewis, F. B. & Saunders, N. H. *J. Phys. C: Solid State Phys.* **6**, 2525 (1973).

[S15] Takeda, M. *et al*. Physical properties of iron-oxide scales on Si-containing steels at high temperature. *Mater. Trans.* **50,** 2242-2246 (2009).
